# Benefits of Adjuvant Mitotane after Resection of Adrenocortical Carcinoma: A Systematic Review and Meta-Analysis

**DOI:** 10.1155/2018/9362108

**Published:** 2018-06-04

**Authors:** Yongquan Tang, Zhihong Liu, Zijun Zou, Jiayu Liang, Yiping Lu, Yuchun Zhu

**Affiliations:** Department of Urology, Institute of Urology, West China Hospital, Sichuan University, Chengdu, China

## Abstract

**Background:**

The adjuvant use of mitotane on adrenocortical carcinoma (ACC) has always been in controversy. We aimed to assess the prognostic benefits of adjuvant mitotane after resection of ACC in patients without distant metastasis.

**Methods:**

The PubMed, WoS, Embase, and Cochrane Library databases were systematically searched. Recurrence-free survival (RFS) and overall survival (OS) were adopted as measurements. A meta-analysis was conducted based on hazard ratio (HR) with 95% confidence interval (CI). A study was included only if the enrolled patients underwent resection of ACC without adjuvant chemotherapy except mitotane.

**Results:**

A total of 5 retrospective studies reporting on 1249 patients were included for this meta-analysis. The meta-analysis showed that adjuvant mitotane was significantly associated with prolonged RFS (HR = 0.62; 95%CI, 0.42-0.94; P < 0.05) and prolonged OS (HR = 0.69; 95%CI, 0.55-0.88, P < 0.05).

**Conclusion:**

After comprehensive review, current evidence suggests that adjuvant mitotane significantly decreases the recurrence rate and mortality after resection of ACC in patients without distant metastasis, but these findings need further demonstration from prospective controlled trials.

## 1. Introduction

Adrenocortical carcinoma (ACC) is a malignancy characterized with much low morbidity (0.5-2/10^∧^6) but very bad prognosis [1]. The 5-year survival rate of patients is approximately 33%-66% in stage I and no more than 5% in stage IV [2]. About 40%-70% of ACCs are functional [3], of which almost 80% are associated with hypersecretion of glucocorticoids (Cushing syndrome). The hypersecretion of androgen, estrogen, or aldosterone is rare [2]. Surgical resection remains the principle strategy for operable ACC, even some local advanced ACC. But the recurrence rate is up to 60%-80% without adjuvant treatment [4, 5].

In 1949, Nelson et al. initially found the adrenolytic effect of 2, 2-di(p'-chlorophenyl)-1,1-dichloroethane (2p'-DDD) in dogs [6]. Ten years later, Bergenstal et al. demonstrated the antitumor effects of 2, 2-di(o, p'-chloropheny)-1,1-dichloroethane (o, p'-DDD), commonly named mitotane, on functional ACC [7]. Mitotane is lipophilic and accumulates primarily in adrenal cortex and adipose tissues [8, 9]. The mechanisms of mitotane against ACC are not fully known. Some researchers found that mitotane and its metabolites could covalently bind to mitochondrial proteins to induce disruption of mitochondria, subsequently resulting in apoptosis of ACC cells [10–13]. Sbiera et al. showed that the inhibition to Sterol-O-Acyl Transferase 1 of mitotane could suppress the synthesis of cholesterol, which would induce excessive accumulation of lipids in endoplasmic reticulum, and then trigger endoplasmic reticulum stress resulting in apoptosis of ACC cells [14]. Recently, Scheidt et al. found mitotane could bind to cell membranes to destroy the membrane integrity [15]. Despite those antitumor efficacies, many patients with ACC had no response to mitotane [16], and the adverse reactions of mitotane also deserve concern. Of patients taking mitotane, approximately 50%-74% have gastrointestinal discomfort [17–19], about 38%-60% may have symptoms in neuromuscular system [17–20], and a minority may present with elevated aminotransferase or alkaline phosphatase, or reduced peripheral blood cells [18, 21]. Besides, mitotane can reduce the synthesis of corticosteroids, leading to adrenocortical insufficiency [22, 23]. Meanwhile, mitotane increases cortisol-binding proteins to reduce free cortisol level [18, 24, 25]. R-Lagunes et al. showed that 17% of patients taking mitotane presented with long-term cortical hypofunction [26].

Given the limited antitumor response and adverse reactions, the efficacy and safety of mitotane have always been in controversy. Besides, the majority of current studies have a small sample size resulting from much low incidence. In patients after tumor resection of ACC, some researchers showed that adjuvant mitotane might improve the recurrence-free survival (RFS) [29, 31], but some others got a negative result [27]. Despite that, mitotane remains the most commonly used therapeutic agent in the treatment of ACC, especially in adjuvant use. This study aimed to include all studies up to date and make a meta-analysis to assess benefit of adjuvant mitotane for postoperative prognosis in patients with ACC.

## 2. Materials and Methods

### 2.1. Search Question

Do adjuvant mitotane after resection of ACC have survival benefits for patients?

### 2.2. Search Strategies

The keywords “mitotane”, “survival”, and “adrenocortical carcinoma or its synonyms” were used to search in PubMed. Then Web of Science, Embase, and Cochrane Library were searched for supplementary. The search was finally updated to September 8, 2017. All records were contained in the literature pool for screening.

### 2.3. Studies Selection

The studies selection was performed by two reviewers independently. The titles and abstracts were firstly viewed to identify unique study enrolling ACC patients with treatment of mitotane. Case reports and nonoriginal publications including editorials, commentaries, and review articles were excluded. Then the full texts of eligible studies were reviewed. References of the selected studies were also checked to search for further eligible studies. We included randomized controlled trials and cohort studies. The studies for inclusion should have comparison of prognoses (RFS or/and overall survival [OS]) of patients with and without adjuvant mitotane after resection of ACC. A study or cohort would be excluded if the enrolled patients had distant metastases of ACC, no resection of ACC, any neoadjuvant therapy before surgery, or adjuvant chemotherapy in addition to mitotane after surgery. But adjuvant radiotherapy was allowed. We only included studies reporting adjusted hazard ratios (HR) in multivariate Cox regression and excluded unadjusted outcome measures because these may provide biased evaluations given the differences in other variates such as age, gender, adjuvant radiotherapy, etc. Duplicates in study cohort were also excluded to leave only one study with the lowest risk of bias.

### 2.4. Risk of Bias Assessment

We used the Newcastle-Ottawa Scale (NOS) for the quality assessment of nonrandomized controlled studies. The follow-up was considered to be adequate if it was over 5 year in median or mean time. Studies with scores ≥ 7 were supposed to have a low risk of bias, scores of 4–6 to have a moderate risk of bias, and scores < 4 to have a high risk of bias [32]. The publication bias was assessed by funnel plots and Begg's test, which was carried out in Stata 14.0 (Stata Corp, College Station, Texas, USA).

### 2.5. Data Extraction

The full texts were carefully reviewed by two reviewers independently. Disagreements were resolved by consensus with the senior author. We collected the following data if available:

1. Study design and quality: publication year, country of patients, study type, patients enrolling criteria, median/maximum of follow-up, and assessed NOS score (Table 1)
2. Multivariable factors: characteristics of cohort and potential prognostic factors adjusted for mitotane in multivariate Cox regression model, including sample size, median or mean age, proportions of male, local advanced cases (stage IV without distant metastasis), positive surgical margins, functional status, and adjuvant radiotherapy (Table 2)
3. Outcome measures: HR of adjuvant mitotane with 95% confidence interval (CI) in RFS and OS respectively, produced by multivariate Cox progression (Table 2)

### 2.6. Statistical Analysis

The heterogeneity was identified by Q test, estimated by DerSimonian-Laird method and quantified by I^2^ values [33]. Given the significant heterogeneity (P < 0.1 or/and I^2^ > 50%) for each analysis, random effects model was employed. Otherwise fixed effect model was employed. Heterogeneity test and meta-analysis were performed by the Review Manager 5.3 (Copenhagen: the Nordic Cochrane Centre, the Cochrane Collaboration, 2014) software. We used the inverse variance method for the meta-analysis of HR. The statistical differences were assessed by Z test. The difference was significant given the P < 0.05.

## 3. Results

### 3.1. Description of Included Studies

**Figure 1:**
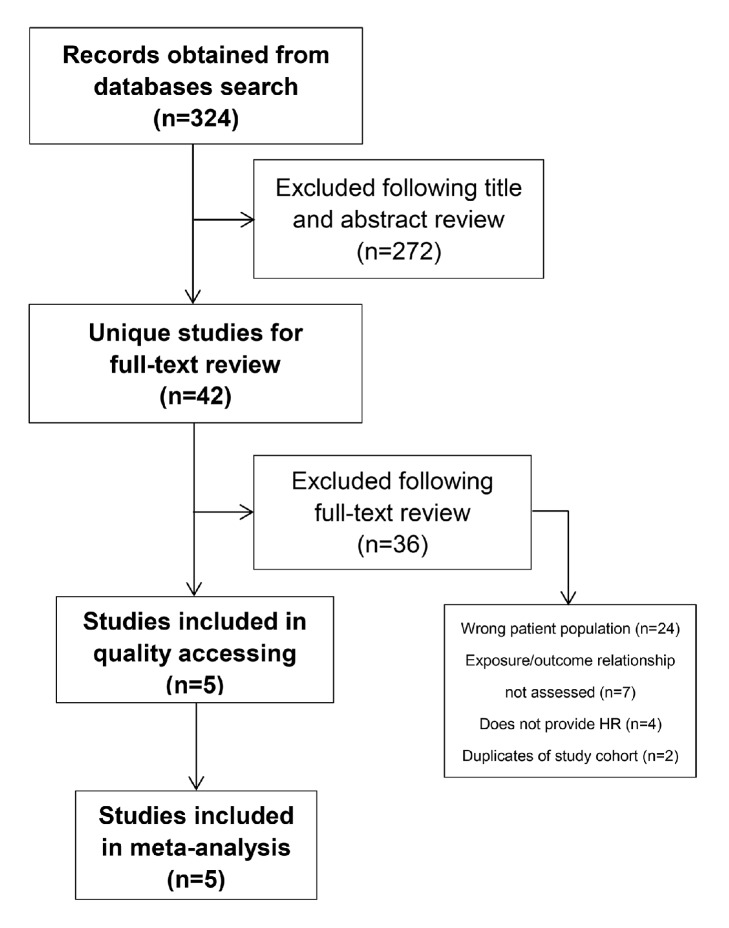
Flow diagram of literature search and studies selection for meta-analysis.

Our literature searching identified 324 records. After full-text review of 42 unique studies, 5 studies were included for meta-analysis. The reasons for exclusion are provided in Figure 1. The detail characteristics of these studies were provided in Table 1. All included studies provided adjusted HR with 95%CI between adjuvant mitotane and nonmitotane in RFS or/and OS. All enrolled patients had resection of ACC without other chemotherapy in addition to mitotane. Patients were enrolled from 1979 to 2014. Overall, 1249 patients were included in the meta-analysis, of which 408 patients (33%) received adjuvant mitotane, 32%-39% were male, and 43%-55% had hormone secreting ACC. Median age was 43-51 years old. Three studies enrolled patients with local advanced ACC (4%-31%) [16, 27, 29]. Three studies provided exact numbers of enrolled patients with positive surgical margins (0%-31%) [27, 29, 30]. And two studies declared that patients with adjuvant radiotherapy were allowed [27, 30] (Table 2).

Among them, the study of Berruti et al. (2017) had 2 independent control groups enrolling Italian (n = 45) and German (n = 70), respectively, relative to a shared mitotane group (n = 47) [16]. The German group was excluded since the patients have been reported before by Fassnacht in the same center (University Hospital of Würzburg, Germany) [30].

### 3.2. Risk of Bias

All included studies were assessed to have a low risk of bias (NOS score ≥ 7) (Table 1). Especially, publication bias of outcomes was not significant (P > 0.05) according to Begg's test.

### 3.3. Recurrence-Free Survival and Overall Survival

**Figure 2:**
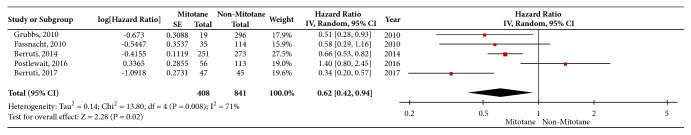
Forest plots of Hazard ratio (HR) on recurrence-free survival (adjuvant mitotane relative to nonmitotane) after resection of adrenocortical carcinoma in patients without distant metastasis. The square data markers represent log [HR] and horizontal lines represent 95% confidence interval (CI) of log [HR]. Marker size reflects the statistical weight of the meta-analysis. The diamond data marker represents the overall log [HR] and 95%CI for the outcome of interest.

**Figure 3:**
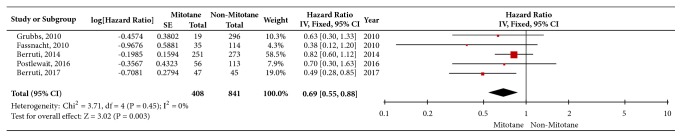
Forest plots of hazard ratio (HR) on overall survival (adjuvant mitotane to nonmitotane) after resection of adrenocortical carcinoma in patients without distant metastasis. The square data markers represent log [HR] and horizontal lines represent 95% confidence interval (CI) of log [HR]. Marker size reflects the statistical weight of the meta-analysis. The diamond data marker represents the overall log [HR] and 95%CI for the outcome of interest.

In total, 5 studies reporting on 1249 patients were included to assess the effect of adjuvant mitotane on RFS. The heterogeneity of outcomes was significant (P = 0.01, I^2^ = 71%). So the random effects model was employed. As a result, adjuvant mitotane was significantly associated with prolonged RFS (HR = 0.62; 95%CI, 0.42-0.94, P = 0.02) (Figure 2). The same crews of studies were included to assess the effect of adjuvant mitotane on OS. There was no evidence of significant heterogeneity among these studies (P = 0.45, I^2^ = 0%). So fixed effect model was employed. As a result, adjuvant mitotane was significantly associated with prolonged OS (HR = 0.69; 95%CI, 0.55-0.88, and P < 0.01) (Figure 3).

## 4. Discussion

This review and meta-analysis identified that adjuvant mitotane was significantly associated with prolonged RFS and OS after resection of ACC in patients without distant metastasis. It indicates that adjuvant mitotane tends to reduce 38% of postoperative recurrences and 31% of postoperative deaths.

To the best of our knowledge, this study represents the first meta-analysis and most up-to-date reviews on this topic. There were only some evidence reviews before on adjuvant use of mitotane. Veytsman et al. claimed that adjuvant mitotane remained controversial although most clinicians agreed that adjuvant mitotane should be used given a high likelihood of recurrence [34]. Campbell-Walsh Urology, 10^th^ Edition, read that few studies demonstrated a significant survival benefit from mitotane as a single agent [2]. Terzolo et al. also did not provide a definite recommendation referring to 3 retrospective studies [35]. All these reviews were limited by few references and lacking meta-analysis of outcomes.

The major strengths of this meta-analysis include systematic search strategies, careful studies selection and data extraction, critical bias assessment, and feasible statistical method. Both NOS and Begg's test indicated that these included studies had a low risk of bias. The age, gender, and functional status among these included studies were very similar (Table 2). Although we found statistically significant heterogeneity of outcomes for RFS analysis, random effects model was employed. Influence-analysis found that Postlewait, 2016 predominated in the heterogeneity. This likely resulted from small cohort size with much more differences in other factors like functional status, stage, postoperative adrenal insufficiency, adjuvant radiotherapy, etc. [27]. Actually, 7 studies were eligible for our aims, but two of them were excluded for duplicating report. Terzolo had the same cohorts with Berruti (2017), and the former was excluded since its shorter follow-up [16, 31]; Berruti (2014) had two common participating centers with Berruti (2017), but it declared that patients included in this study had not been included in the study of Terzolo that had the same cohorts with Berruti (2017) [16, 28]. The study of Else enrolled patients in University of Michigan Hospital from 1979 to 2013, which have been reported by Berruti (2014), and the former was excluded since its smaller sample size [28, 36].

A key limitation is that this meta-analysis is primarily based on retrospective studies, since no fully prospective controlled study is available to our knowledge. So potential biases deserved concerns on selection bias, lost to follow-up, confounding factors, and reporting bias etc. Firstly, it is known that adjuvant mitotane tended to be used in patients with a high likelihood of recurrence [34] given infiltration out of adrenal, local lymph nodes metastasis, and/or positive surgical margins, which may produce selection bias in each study. Despite that, the RFS and OS in mitotane cohort were still significantly superior to that in nonmitotane cohort, which provided further support to our findings. Secondly, Kaplan-Meier survival rate curve could largely reduce bias of loss, and all included studies had follow-up over 5 years if not death or loss. Thirdly, positive surgical margins and adjuvant radiotherapy may be potential confounding factors. Postlewait et al. said that both microscopically and macroscopically positive margins were significantly independent risk factors for OS [27]. But several studies revealed that adjuvant radiotherapy was not associated with RFS and OS in adjusted HR analysis [27, 36, 37]. Although we were not able to make subgroup analysis to explore these two potential confounding factors since insufficient data, all extracted HR with 95%CI have been adjusted to other potential prognostic factors in multivariate Cox regression. The salvage treatment after recurrence was another potential prognostic factor for OS. Three studies declared that patients received salvage surgery, radiotherapy, and/or chemotherapy after recurrence, but all of them did not consider it as an adjusting factor [16, 27, 30]. However, all patients in a certain center or country were generally managed following common criteria despite whether a patient received adjuvant mitotane or not. In addition, some other researchers indicated that maintenance of mitotane concentrations ≥ 14 mg/L was significantly associated with RFS benefit [38]. But plasma level of mitotane was rarely revealed in these included studies, most likely because few data were available [27, 36]. Fourth, postoperative death was not clearly defined either by ACC-specific death or by all-course death by authors except Fassnacht et al. [27]. To reduce this bias, Berruti et al. (2017) excluded patients with clinically significant concomitant diseases [16]. Towards benefit for patients, it is obvious that RFS and OS cannot represent all. Regarding potential side effects, the quality-of-life during adjuvant mitotane should be considered, but no study has assessed it. While generally, most clinicians agreed that mitotane was used only if well tolerated [39].

Briefly, this study provides very comprehensive evidences, and most potential biases were controlled. Therefore we recommend adjuvant use of mitotane after resection of ACC if well tolerated, because adjuvant mitotane was associated with significantly prolonged RFS and OS on these patients.

## 5. Conclusion

Mainly based on retrospective cohort studies, this meta-analysis suggests that adjuvant mitotane significantly decreases recurrence rate and mortality after resection of ACC in patients without distant metastasis, but this finding needs further demonstration from prospective controlled trials.

## Figures and Tables

**Table 1:** Characteristics of included studies and quality assessing of non-randomized studies with Newcastle-Ottawa Scale (NOS).

Reference	Year	Country	Inclusion criteria^a^	Follow-up^b^	Adjusting factors^c^	NOS
Berruti [16]	2017	Italy	1985-2003 ≥ 18 years old R0, R1 Stage I-IV No radiotherapy	>128/>200 months	Age Gender Stage	8
Postlewait [27]	2016	USA	1993-2014 R0, R1,R2 Stage I-IV Allowed radiotherapy	44/>60 months	Age Function Stage Surgical margins Radiotherapy et al.	7
Berruti [28]	2014	Italy Germany Holland France USA	1990-2008 ≥ 18 years old R0 Stage I-IV Allowed radiotherapy	50/>120 months	Age Gender Function Stage	7
Grubbs [29]	2010	USA	1991-2008 R0, R1, Rx Stage I-IV	88/>100 Months	Age Gender Function Stage Institution	8
Fassnacht [30]	2010	Germany	1990-2009 R0, R1, R2, Rx Stage II Allowed radiotherapy	38/>60 months	Surgical margins Mitotic count	7

a R0 denotes microscopically negative margin; R1 denotes microscopically positive margin; R2 denotes grossly positive margin.

b Median/maximum length of follow-up.

c Prognostic factors adjusted for mitotane in multivariate Cox regression model.

**Table 2:** Characteristics of patients and hazard ratios (HR) (95% confidence interval [95%CI]) of mitotane in multivariate Cox regression model fitted for time of recurrence-free survival (RFS) and overall survival (OS), adjusted by age, gender, stage, positive surgical margins (PSMs), functional status and/or adjuvant radiation, etc.

Reference	Year	Total No.	Age/y^a^	Male/%	Stage IV/%^b^	PSMs/%^c^	Function/%	Radiation/%	Mitotane^d^	RFS/HR(95%CI)^e^	OS /HR(95%CI)^e^
Berruti [16]	2017	92	43	32	11	-	45	0	47	1/2.98(1.75-5.09)	1/2.03(1.17-3.51)
Postlewait [27]	2016	169^g^	51	39	31	31	43	10	56	1.4(0.8-2.4)	0.7(0.3-1.5)
Berruti [28]	2014	524	45	39	0	0	52	-	251	0.66(0.53-0.83)	0.82(0.60-1.10)
Grubbs [29]	2010	215	46	35	4	27	55	-	19	1/1.96(1.07-3.61)	1/1.58(0.75-3.31)
Fassnacht [30]	2010	149	48	35	0	9	-	9	35	0.58(0.29-1.15)	0.38(0.12-1.28)

a Median or mean age of total population.

b Local advanced cases without distant metastasis.

c (R1+R2) / (R0+R1+R2) (R0 denotes microscopically negative margin; R1 denotes microscopically positive margin; R2 denotes grossly positive margin) in spite of Rx or data loss.

d Number of patients who received mitotane.

e HR of mitotane relative to nonmitotane. When a study provided HR of no mitotane relative to mitotane, it was recorded as 1/HR.

## Additional Points

A key publication error in an included study was confirmed by its corresponding author (Maithel, S. K.) that “adjuvant chemotherapy” in last row of the table in page 8 should actually be read “mitotane.”
